# Treatment of a schizophrenia patient on long-term super-dose antipsychotics: a case report

**DOI:** 10.3389/fpsyt.2024.1533350

**Published:** 2025-01-20

**Authors:** Xiaobo Xie, Jinzhang Chen, Hongli Song, Zebin Fan

**Affiliations:** Department of Psychiatry, The Fifth People's Hospital of Xiangtan City, Xiangtan, China

**Keywords:** clozapine, chlorpromazine, antipsychotics, schizophrenia, case report

## Abstract

**Introduction:**

Clozapine and chlorpromazine are widely used for treating schizophrenia. However, irregular medical follow-ups are common in patients with schizophrenia, potentially leading to long-term super-dose medication. Managing such cases poses significant challenges for clinical psychiatrists.

**Case presentation:**

This report describes a 24-year-old Han Chinese male diagnosed with schizophrenia who had been taking long-term super-doses of clozapine (18–107 tablets/day, 25 mg/tablet) and chlorpromazine (7–40 tablets/day, 50 mg/tablet) for five months due to irregular medical follow-ups. Upon hospitalization, the doses of antipsychotic drugs were gradually tapered, and the medication regimen was adjusted based on the patient’s previous treatment history. Comprehensive health education about schizophrenia was also provided. The patient was followed for four years, during which his psychiatric symptoms remained under partial control.

**Conclusion:**

Clinicians must consider individual differences in the efficacy and adverse effects of antipsychotics and weigh the benefits and risks of combination therapy. Future efforts should focus on strengthening health education for patients with schizophrenia and their families to improve treatment compliance and outcomes.

## Introduction

Chlorpromazine and clozapine are effective treatments for schizophrenia, and their relatively low cost contributes to their widespread use in China. Previous case reports have documented many instances of single-drug overdose with clozapine or chlorpromazine, often due to suicide attempts or accidental ingestion (1, 2). However, cases involving the long-term simultaneous use of both antipsychotics at super-doses over several months are rare. Managing schizophrenia in patients with prolonged super-dose polypharmacy presents significant challenges for clinicians.

Herein, we report a case involving a schizophrenia patient who self-administered exceedingly super-doses of clozapine and chlorpromazine for an extended period due to nonadherence to medical advice. We describe the clinical experience of treating this patient, including dose reduction, drug withdrawal, combined use of risperidone to enhance efficacy, supportive treatment for physical conditions, and health education for the patient and their family improve the patient’s adherence and outcomes. Consent to publish the case history was obtained from the patient. The Fifth People’s Hospital of Xiangtan City approved the study protocol.

## Case presentation

A 24-year-old male patient of Han ethnicity was admitted to the psychiatric ward of the Fifth People’s Hospital of Xiangtan City on July 14, 2020, presenting with a seven-year history of schizophrenia and a five-month history of intermittent paroxysmal sialorrhea and convulsions. These symptoms emerged following self-administration of clozapine (18–107 tablets/day, 25 mg/tablet) and chlorpromazine (7–40 tablets/day, 50 mg/tablet). Before this admission, the outpatient physician explicitly informed the patient’s family that the patient’s condition was critical, with a potential risk of life-threatening complications at any moment, and strongly recommended immediate transfer to the emergency department for further evaluation and necessary auxiliary examinations. However, the patient’s family insisted on admission to the psychiatric ward, citing financial constraints. After signing an informed consent form acknowledging the associated risks and accepting full responsibility for any potential adverse outcomes, the psychiatric department proceeded with the patient’s admission.

### History of present illness

**Figure 1:**
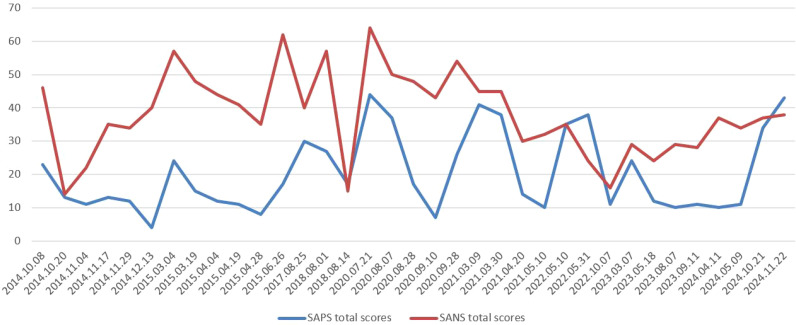
Timeline of SAPS and SANS total scores from 2014 to 2024.

Based on the ICD-10 diagnostic criteria, the patient was diagnosed with schizophrenia in 2013 after exhibiting social withdrawal, auditory hallucinations (perceiving voices without an external source) and laughing to himself without apparent cause for six months. Initial treatment with risperidone (4 mg/day) did not achieve complete symptom resolution. Between 2014 and 2018, he was admitted to the hospital five times, during which risperidone was combined with clozapine, partially stabilizing his positive symptoms. However, his negative symptoms remained significant (for details, see **Figure 1**, **Tables 1**–**3**). Despite regular medication adherence post-discharge, he remained indolent and socially withdrawn, unable to study or work from 2018 to 2020.Within the five months before the hospitalization on July 14, 2020, the patient and his family deviated from medical advice, escalating the daily dose of clozapine to 18-107 tablets (25mg/tablet) and chlorpromazine to 7-40 tablets (50mg/tablet). The patient had taken 107 tablets/day of clozapine and 40 tablets/day of chlorpromazine for more than half a month. Prior to admission, the doses had been adjusted to 32 tablets/day of clozapine and 9 tablets/day of chlorpromazine. During this period, the patient experienced intermittent convulsions and episodes of unconsciousness lasting 5–6 minutes, which resolved spontaneously. Despite these events, no medical attention was sought. On July 14, 2020, the patient demonstrated severe psychomotor retardation and mental sluggishness, prompting hospitalization.

**Table 1:** Timeline of taking medication.

Number of hospitalizations	Duration of hospitalizations	Clozapine	Risperidone	Chlorpromazine	Aripiprazole	Perphenazine	Magnesium Valproate Sustained-Release Tablets
1	2014.10.08-2014.12.09	125mg/d	4mg/d	–	–	–	–
2	2015.03.03-2015.04.29	75mg/d	5mg/d	–	–	–	–
3	2015.06.25-2015.07.04	50mg/d	5mg/d	–	–	–	–
4	2016.07.13-2016.08.16	125mg/d	5mg/d	–	–	–	–
5	2018.07.23-2018.08.21	50mg/d	3mg/d	–	–	–	–
–	2020.2-2020.07.14	450mg-2675mg/d	–	350mg-2000mg/d	–	–	–
6	2020.07.14-2020.10.12	200mg/d	5mg/d	–	–	–	1g/d
7	2021.02.22-2021.05.17	200mg/d	5mg/d	–	–	–	1g/d
8	2022.05.09-2022.06.08	100mg/d	–	–	30mg/d	–	0.5g/d
9	2022.09.28-2022.10.09	100mg/d	–	–	30mg/d	–	0.5g/d
10	2023.03.07-2023.04.13	225mg/d	–	–	–	10mg/d	0.5g/d
11	2023.05.08-2023.08.09	250mg/d	5mg/d	–	–	–	0.5g/d
12	2023.08.21-2024.02.05	200mg/d	4mg/d	–	–	–	0.5g/d
13	2024.03.25-Now	200mg/d	4mg/d	–	–	–	0.5g/d

**Table 2:** Timeline of SAPS scores from 2014 to 2024.

Assessment Time	Total scores	Comprehensive Assessment Score	Hallucinations	Delusions	Bizarre Behavior	Positive Formal Thought Disorder
2014.10.08	23	7	3	3	1	0
2014.10.20	13	5	3	0	2	0
2014.11.04	11	5	2	1	2	0
2014.11.17	13	5	3	1	1	0
2014.11.29	12	2	2	0	0	0
2014.12.13	4	1	0	0	1	0
2015.03.04	24	8	3	3	1	1
2015.03.19	15	5	3	1	0	1
2015.04.04	12	4	3	1	0	0
2015.04.19	11	3	2	1	0	0
2015.04.28	8	3	2	1	0	0
2015.06.26	17	8	4	4	0	0
2017.08.25	30	4	2	2	0	0
2018.08.01	27	8	3	3	1	1
2018.08.14	17	4	2	2	0	0
2020.07.21	44	8	3	3	1	1
2020.08.07	37	4	1	1	1	1
2020.08.28	17	4	2	0	1	1
2020.09.10	7	2	1	1	0	0
2020.09.28	26	4	1	1	1	1
2021.03.09	41	7	2	2	2	1
2021.03.30	38	5	2	1	1	1
2021.04.20	14	4	2	0	1	1
2021.05.10	10	3	2	0	0	1
2022.05.10	35	7	3	3	1	0
2022.05.31	38	9	3	2	2	2
2022.10.07	11	5	2	1	2	0
2023.03.07	24	9	4	1	3	1
2023.05.18	12	4	1	1	1	1
2023.08.07	10	4	1	1	1	1
2023.09.11	11	3	1	1	1	0
2024.04.11	10	3	1	1	1	0
2024.05.09	11	3	1	1	1	0
2024.10.21	34	5	2	1	1	1
2024.11.22	43	7	3	1	2	1

**Table 3:** Timeline of SANS scores from 2014 to 2024.

Assessment Time	Total scores	Comprehensive Assessment Score	Anhedonia	Alogia	Avolition	Asociality	Attention
2014.10.08	46	10	3	0	3	3	1
2014.10.20	14	4	2	0	2	0	0
2014.11.04	22	4	2	0	2	0	0
2014.11.17	35	6	2	0	2	2	0
2014.11.29	34	7	2	0	2	2	1
2014.12.13	40	8	2	0	2	2	2
2015.03.04	57	15	3	3	3	3	3
2015.03.19	48	12	2	2	3	3	2
2015.04.04	44	12	2	2	3	3	2
2015.04.19	41	11	2	2	3	2	2
2015.04.28	35	11	2	2	2	3	2
2015.06.26	62	14	3	2	3	3	3
2017.08.25	40	11	2	2	3	2	2
2018.08.01	57	13	3	3	3	3	1
2018.08.14	15	3	1	0	1	1	0
2020.07.21	64	15	3	3	3	3	3
2020.08.07	50	11	3	1	3	3	1
2020.08.28	48	11	3	2	2	3	1
2020.09.10	43	9	3	1	2	2	1
2020.09.28	54	11	3	1	3	2	2
2021.03.09	45	9	2	2	2	2	1
2021.03.30	45	9	2	2	2	2	1
2021.04.20	30	8	2	2	2	2	0
2021.05.10	32	8	2	1	2	2	1
2022.05.10	35	10	2	1	3	3	1
2022.05.31	24	5	1	1	1	1	1
2022.10.07	16	4	0	0	2	2	0
2023.03.07	29	9	2	0	3	2	2
2023.05.18	24	8	1	0	2	2	3
2023.08.07	29	7	2	1	1	2	1
2023.09.11	28	8	0	2	2	2	2
2024.04.11	37	8	2	0	2	3	1
2024.05.09	34	7	2	1	2	2	0
2024.10.21	37	9	2	1	3	2	1
2024.11.22	38	10	2	2	3	2	1

### Past medical history

The patient had no known drug allergies but sustained a spinal injury in 2015 following a suicide attempt. He underwent surgical intervention and recovered well.

### Personal history

The patient, born of a normal pregnancy and natural delivery, attended a vocational college in China but drop out of school, exhibited poor academic performance and social functioning after the onset of schizophrenia. He neither smoked nor consumed alcohol and reported no history of substance abuse or exposure to infested water. Between 2014 and 2020, the patient’s positive symptoms were partially controlled, but negative symptoms persisted and fluctuated, significantly impairing his social functioning. The patient’s parents had limited education and lacked basic knowledge about the mental disorder. Additionally, the family’s poor financial situation made it difficult to afford regular medical visits. Despite this, the family had high expectations for the patient’s treatment, hoping he could return to work rather than remain socially withdrawn at home. Consequently, during early 2020, they chose not to follow regular medical appointments and instead increased the patient’s medication dosage on their own, ultimately leading to the patient’s long-term super dose.

### Marital history

Unmarried.

### Family history

The patient’s mother was suspected of having an undiagnosed mental illness.

## Clinical findings

### Physical examination

#### Vital signs

Temperature: 36.6°C; Pulse: 122 bpm; Respiratory Rate: 20 breaths/min; Blood Pressure: 130/86 mmHg

#### Physical findings

Body weight: 52 kg; Height: 165 cm. The patient exhibited excessive drooling, lethargy, and no obvious skin rash. Heart rate was 122 bpm, lung sounds were clear, and the abdomen was soft.

#### Mental status examination

The patient was accompanied by family members and required assistance to complete the hospital admission process. He lacked insight and appeared unkempt, with impaired consciousness (lethargy). He could be awakened by calling and was able to answer simple questions, although some responses were irrelevant. Once stimulation ceased, he quickly returned to sleep. He denied hallucinations but reported paranoid ideation and poor attention. Emotional stability was preserved, but volitional activity was markedly diminished. He denied any impulsive behaviors, such as harming others or destroying property. He spent most of his time lying in bed, exhibited minimal social interaction, and remained largely isolated.

#### The outpatient auxiliary examination

Chest CT revealed no abnormalities.

### Diagnostic assessment

Based on the patient’s history and physical examination, the provisional diagnoses were: 1) Harmful effects of drugs and agents, suspected drug poisoning; 2) Extrapyramidal adverse reactions; 3) Schizophrenia; 4) Sinus tachycardia.

Routine blood tests showed leukocytosis (WBC: 13.82 × 10⁹/L, neutrophils: 10.42 × 10⁹/L). The electrocardiogram revealed sinus tachycardia. Tests for N-terminal pro-brain natriuretic peptide (NT-proBNP), procalcitonin, troponin T, liver and kidney function, blood lipids, blood glucose, C-reactive protein, coagulation function, myocardial enzymes, thyroid function, and electrolytes showed no significant abnormalities. Additionally, screenings for syphilis, HIV, hepatitis B, and hepatitis C were negative. Imaging studies, including a CT head plain scan, electroencephalogram, and color Doppler ultrasound of the abdominal and urinary systems, also revealed no notable findings.

### Therapeutic interventions

#### Initial management

**Figure 2:**
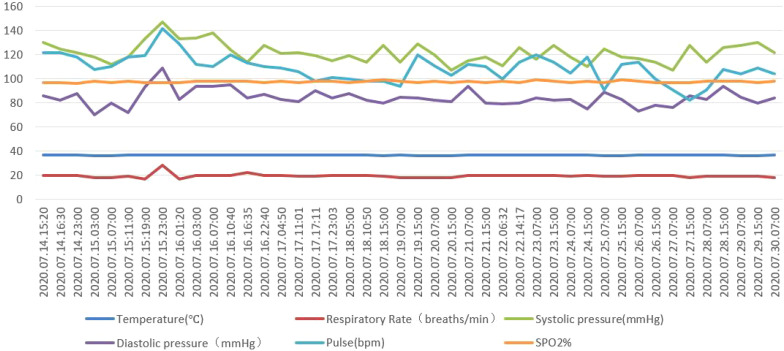
Timeline of vital signs from 2020.07.14 to 2020.07.30.

During the first week of hospitalization, vital signs were monitored (for details, see **Figure 2**), and symptomatic treatments were initiated to enhance drug excretion. Clozapine was reduced from 32 tablets/day (25 mg/tablet) to 250 mg/day, and chlorpromazine was discontinued. During the period of drug reduction and withdrawal, fluctuations in psychiatric symptoms were closely monitored. Benzhexol, at a dose of 4 mg/day, was administered to mitigate extrapyramidal side effects, and magnesium valproate sustained-release tablets, at 1 g/day, were prescribed to prevent seizures. Following these treatments, the patient’s vital signs remained stable under continuous monitoring.

#### Subsequent management

In the second week of hospitalization, the patient’s consciousness fully recovered, and the dose of clozapine was reduced to 175 mg/day. However, the patient’s psychotic symptoms fluctuated. On July 21, 2020, the Scale for Assessment of Positive Symptoms (SAPS) was used to evaluate positive symptoms, yielding a total score of 44. The Scale for Assessment of Negative Symptoms (SANS) was used to assess negative symptoms, with a total score of 64 (for details, see **Figure 1**, **Tables 2**, **3**). Treatment was initiated by gradually increasing the dose of risperidone, which had previously proven effective for this patient, to 3 mg/day as a synergistic approach to managing psychotic symptoms. The doses of clozapine and risperidone were dynamically adjusted throughout the hospitalization.

During the subsequent hospitalization, the patient was treated with clozapine at 200 mg/day in combination with risperidone at 5 mg/day, resulting in a gradual partial alleviation of psychotic symptoms. The patient’s physical condition stabilized, and follow-up examinations of the previously assessed indicators revealed no significant abnormalities. During this period, the medical staff provided structured and progressive health education about schizophrenia, the appropriate use of antipsychotic medications, and treatment outcome expectations, tailored to the patient and family’s financial and educational circumstances. Simultaneously, the medical team worked collaboratively with the patient, inquiring about his treatment preferences and incorporating his perceptions of medication into clinical considerations to improve adherence. Additionally, guidance extended to post-discharge follow-up, the importance of timely medical consultations, and applicable social security policies, ensuring that the patient and family understood when and how to seek assistance. By the time of discharge, the patient was fully conscious, and follow-up tests—including routine blood tests, liver and kidney function tests, cardiac enzyme assays, lipid profile, electrolytes, fasting blood glucose, and electrocardiogram—showed no significant abnormalities. The positive and negative symptoms of the patients were partially improved (for details, see **Tables 2**, **3**, and **Figure 1** for the medical records from July 21, 2020, to September 28, 2020).

#### Discharge and follow-up

After completing the health education program and receiving relevant guidance during this hospitalization, the patient committed to adhering to the prescribed regimen, while the family pledged to encourage and support regular follow-up appointments. Upon discharge on October 12, 2020, the patient was prescribed clozapine (200 mg/day), risperidone (5 mg/day), and magnesium valproate (1 g/day).

From 2020 to 2024, although no severe overdosing incidents occurred as before, the patient was readmitted multiple times due to being unattended at home and lapses in adherence. Adjustments to the treatment regimen demonstrated that the combination of clozapine and risperidone consistently yielded better therapeutic outcomes and medication adherence than other treatment regimens (see **Tables 1**–**3**, and **Figure 1** for details on medication and the severity of psychiatric symptoms). In addition, there were no significant abnormalities observed in the patient’s physical examination or laboratory reexaminations. During these hospitalizations, various treatment regimens, including clozapine/aripiprazole and clozapine/perphenazine, were trialed to manage his psychotic symptoms; however, the effectiveness was not as favorable as with the clozapine/risperidone combination. From May 2023 to the present, the patient has continued treatment with clozapine/risperidone to manage his psychiatric symptoms, and his family members formally declined further modified electroconvulsive therapy. Currently, the patient remains in extended care at our hospital but is able to manage daily activities independently.

## Discussion

The prolonged use of super-dose antipsychotic combinations, as observed in this case, underscores the challenges in managing treatment adherence in schizophrenia. Contributing factors are strongly related to the characteristics of the schizophrenia population, including low education levels (3), insufficient mental health literacy (4), low socioeconomic status (5), complex family backgrounds (such as single, divorced, widowed, or left-behind individuals), superstition, and a low awareness and treatment willingness among patients and their families regarding mental illness. These factors can easily lead to delays in seeking care and aggravation of the disease. Even after the first visit, some patients and their families exhibit irregular follow-up, blindly increase the doses of antipsychotic drugs, and often mistake adverse reactions for psychiatric symptoms, leading to further unnecessary dose increases. Due to the lack of follow-up guidance from healthcare professionals, these patients often show no improvement in psychiatric symptoms after prolonged use of super-doses of antipsychotic drugs, and they experience significant physical adverse reactions.

For patients on long-term, super-dose antipsychotic medication, ensuring their safety and minimizing potential adverse physical consequences are of paramount importance. During the diagnosis and treatment, it is essential to gather a thorough medication history, including information about allergies and the specifics of antipsychotic use (such as the drug name, dose, and duration). The patient’s vital signs and physical symptoms should be closely monitored. If the patient’s level of consciousness is significantly impaired or if vital signs are unstable, it is advisable to admit them to the emergency department for further evaluation and appropriate diagnostic tests, thereby minimizing potential risks and adverse outcomes. Moreover, complete symptomatic and supportive treatment should be considered. Specifically, due to the high lipid solubility of clozapine, conventional treatments such as diuretics or hemodialysis are ineffective for clozapine poisoning. Previous study has suggested implementing hemoperfusion therapy when feasible (6) alongside continuous monitoring of blood drug concentrations. Maintaining stable vital signs is crucial, but the challenge remains in how to further adjust treatment strategies for these patients, and this issue is still a matter of debate.

From the perspective of antipsychotic drug adverse reactions, long-term use of super doses may lead to tardive dyskinesia, increased prolactin levels, toxic liver damage, bone marrow suppression, seizures, allergic rashes, exfoliative dermatitis, and neuroleptic malignant syndrome (7). However, in this case, the patient’s vital signs and laboratory results showed no significant clinical abnormalities, even after five months of super-dose treatment with both clozapine and chlorpromazine prior to hospitalization. This suggests considerable individual variability in the adverse reactions to antipsychotic drugs. The patient’s family reported that the patient had experienced several epileptic episodes during the super-dose medication phase before hospitalization. Additionally, regarding the antipsychotic drug withdrawal in patients taking long-term super doses, previous case studies have shown that rapid withdrawal from large doses of clozapine can lead to seizures (8). Therefore, in this case, magnesium valproate sustained-release tablets (1g/day) were used to prevent seizures, which is consistent with previous case reports (9).

In terms of adjusting treatment strategies to achieve better therapeutic outcomes and thereby avoid blindly increasing antipsychotic dosages, some hospitals have begun to use genetic analysis of antipsychotic drug responses for schizophrenia patients who have failed to respond to empirical treatments. By analyzing genetic differences in combination with drug concentration testing and long-acting injectable antipsychotics, treatment plans are optimized. However, due to the lack of relevant testing equipment and the relatively high cost, this approach has not been widely implemented in most psychiatric hospitals in China. Currently, treatment strategies primarily rely on the patient’s medication history, physical examination, and necessary laboratory tests, with decisions based on the clinician’s experience with single or combined antipsychotic drugs. The combination of two antipsychotic drugs for the treatment of schizophrenia is a complex and challenging strategy, with the potential advantages of covering a broader range of psychiatric symptoms through the different mechanisms of action of the drugs. This can reduce the dose of each drug, potentially minimizing the side effects of a single drug. However, combining drugs also increases the risk of side effects and drug interactions, which may raise the treatment burden, reduce patient compliance, and lead to overdosing or missed doses. To support conventional treatment strategies, psychiatric hospitals should place greater emphasis on strengthening health education for patients with mental disorders and their families to improve medication adherence and self-management skills (10). Implementing targeted health education tailored to the educational and economic circumstances of patients and their families is particularly crucial for achieving these goals. Achieving an effective and safe treatment strategy requires careful monitoring of the patient’s therapeutic outcomes, physical condition, and treatment adherence to minimize risks while maximizing therapeutic benefits.

For psychiatric clinicians, balancing the efficacies, adverse reactions, and treatment adherence in patients with refractory schizophrenia necessitates adopting further individualized treatment strategies tailored to each patient’s overall condition and medical history. These strategies often involve the combined use of antipsychotic drugs, along with repeated adjustments and testing to determine the most suitable treatment regimen. Patients and their families should be fully informed of the potential risks and benefits and collaboratively involved in the decision process to enhance adherence. Previous studies have reported evidence supporting the use of clozapine combined with risperidone (11, 12), clozapine combined with aripiprazole (13, 14), and clozapine combined with first-generation antipsychotics (15) in patients with refractory schizophrenia who do not achieve adequate symptom control with monotherapy. Drawing on these findings and the patient’s suboptimal therapeutic response, we attempted three combination therapies during follow-up: clozapine plus risperidone, clozapine plus aripiprazole, and clozapine plus perphenazine. A closer examination of the patient’s psychiatric symptom scores (SANS and SAPS) revealed that periods with both scores below 20 occurred mainly on three periods: October 20, 2014 (clozapine plus risperidone), August 14, 2018 (clozapine plus risperidone), and October 7, 2022 (clozapine plus aripiprazole). Further analysis of the medical history suggests that after October 20, 2014 and August 14, 2018, the patient’s psychiatric symptoms worsened mainly due to irregular medication adherence post-discharge. Following October 7, 2022 (clozapine and aripiprazole), the patient’s symptoms improved only briefly (SAPS and SANS <20), and despite continued adherence to the regimen, by March 7, 2023, both positive and negative symptoms had again worsened. This finding indicates that the clozapine-plus-aripiprazole combination may not maintain long-term control of both positive and negative symptoms. Moreover, previous literature suggests that considering the patient’s medication preference can enhance treatment adherence (16). During treatment, the patient expressed a preference for the clozapine-plus-risperidone combination over other regimens, perceiving its side effects to be relatively mild. Taken together, continuing the clozapine-plus-risperidone combination was deemed the most appropriate approach for this patient’s treatment. However, it is worth noting that over the past six months (March 25, 2024 to the present), the patient’s positive and negative symptoms have once again shown an upward trend, despite long-term inpatient treatment with the clozapine-plus-risperidone regimen. This suggests that further adjustments in drug selection and dosage may be necessary. In the process of repeatedly adjusting treatment strategies for patients with refractory schizophrenia to develop individualized treatment regimens, a stronger therapeutic alliance was established. This approach improved their understanding of the mental disorder and treatment outcomes, supported by targeted health education, thereby enhancing adherence and cooperation. Even when the therapeutic effect was not as expected, it helped prevent the recurrence of super-dose medication use, ensuring patient safety, minimizing potential adverse consequences, and promoting the possibility of full recovery in the future.

This case has limitations in the treatment process. Due to a lack of equipment, the patient’s blood drug concentration was not measured. Furthermore, medication adjustments were made primarily based on prior experience, though genetic testing could be integrated into future treatment adjustments.

## Conclusion

This case report discusses the treatment of a schizophrenic patient who was on a long-term, super-dose combination of clozapine and chlorpromazine, contributing to valuable clinical experience. Furthermore, psychiatric hospitals should not only focus on providing effective medical treatment but also emphasize strengthening health education on mental illness for patients and their families. Moving forward, we will continue to monitor the patient’s condition closely and develop individualized treatment plans to achieve the best possible therapeutic outcomes.

## Data Availability

The original contributions presented in the study are included in the article/supplementary material. Further inquiries can be directed to the corresponding author.
